# P-829. Determination of novel clinical phenotypes among sepsis patients from an Indian sepsis registry

**DOI:** 10.1093/ofid/ofae631.1021

**Published:** 2025-01-29

**Authors:** Fabia Edathadathil, Georg Gutajhr, T S Dipu, Sanjeev Singh

**Affiliations:** Amrita Institute of Medical Sciences, Kochi, Kerala, India; Amrita Vishwa Vidyapeetham, Kollam, Kerala, India; Amrita Institute of Medical Sciences and Research Center, Kochi, Kerala, India; Amrita Institute of Medical Sciences, Kochi, Kerala, India

## Abstract

**Background:**

Sepsis is a dysregulated host immune response with heterogenous clinical presentation reflected in its high dimensional data and with distinct epidemiological profile endemic to LMICs such as India. The study aims to identify and estimate the prevalence of clinical phenotypes from retrospective data of sepsis patients from a South Indian registry.

Characteristics of the identified clusters and outcomes

**Figure:**
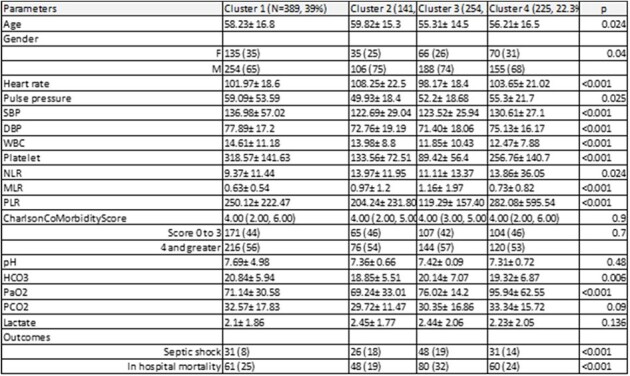

**Methods:**

The registry enrolled all adult patients admitted through the emergency department with a presumed diagnosis of sepsis following Surviving Sepsis Guidelines(SSG) criteria(Edathadathil F, 2022)^1^. Following Ordering points to identify the clustering structure(OPTICS) assessment, k-means clustering was identified and employed as unsupervised clustering method for deriving clusters. The association of focus of infection and outcomes among the clusters was assessed. The identified Indian clusters were compared to the phenotypic clusters derived by Seymour et al using distance matching^2^(Seymour et al, 2019).

Distribution of focus of infection and causative pthogens among the clusters

**Figure:**
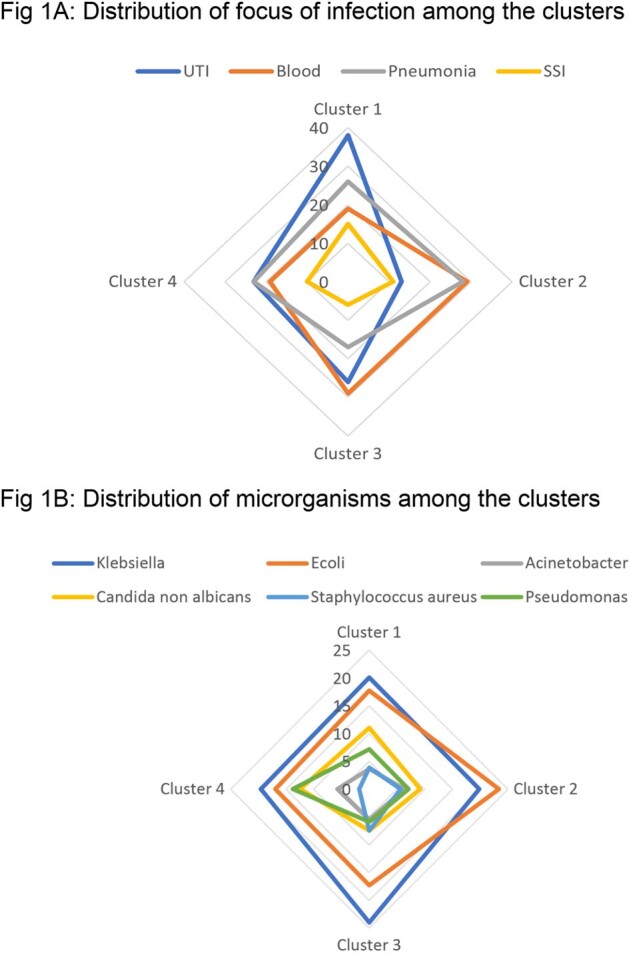

**Results:**

Of the 1009 patients enrolled in the registry, four clusters were identified with Cluster 1 exhibiting the highest prevalence and comprising 389 patients (39%), followed by Cluster 3 (254, 25%), Cluster 4 (225, 22.3%), and Cluster 2 (141, 14%). Age(p=0.024) and gender(p=0.04) significantly differed among the four clusters with septic shock (p< 0.001) and inhospital mortality (p< 0.001) significantly higher in Cluster 3 (septic shock at 19%, in hospital mortality at 32%)(Table 1). Among focus of infection, the prevalence of pneumonia (p=0.018), SSI(p=0.011) and bacteremia(p=0.004) significantly differed among the clusters (Fig 1). The proportion of bacteremia(29%) and pneumonia(28%) were observed to be significantly high in cluster 2. UTI(139, 38%) and SSI (57, 15%) were found to be significantly high in cluster 1. Pseudomonas (p=0.008) and staphylococcus aureus (p=0.02) were observed to be significantly different among the clusters. Clusters 1,2,3 and 4 exhibited close association to the gamma, beta, alpha and delta clusters respectively among the phenotypes derived by Seymour et al.

**Conclusion:**

Our study has been one of the pioneer initiative in demonstrating inherent subgroups among sepsis patients within an Indian cohort data that significantly differed in outcomes and epidemiology.

**Disclosures:**

**All Authors**: No reported disclosures

